# Single-cell atlas of murine adrenal glands reveals immune-adrenal crosstalk during systemic *Candida albicans* infection

**DOI:** 10.3389/fimmu.2022.966814

**Published:** 2022-11-01

**Authors:** Kai Zhang, Yuzhe Hu, Ruoyu Li, Ting Li

**Affiliations:** ^1^ Department of Dermatology and Venerology, Peking University First Hospital, Beijing, China; ^2^ National Clinical Research Center for Skin and Immune Diseases, Beijing, China; ^3^ Research Center for Medical Mycology, Peking University, Beijing, China; ^4^ Beijing Key Laboratory of Molecular Diagnosis on Dermatoses, Beijing, China; ^5^ Department of Immunology, School of Basic Medical Sciences, Peking University Health Science Center, Beijing, China; ^6^ Key Laboratory of Medical Immunology, National Health Commission of the People's Republic of China, Beijing, China; ^7^ Peking University Center for Human Disease Genomics, Beijing, China

**Keywords:** fungal sepsis, systemic *Candida albicans* infection, adrenal gland, single-cell RNA sequencing, immune-adrenal crosstalk

## Abstract

Fungal sepsis remains a major health threat with high mortality, where the adrenal gland stress response has been rarely reported. *Candida albicans* (*C.albicans*) is the most common opportunistic fungal pathogen of life-threatening disseminated candidiasis and fungal sepsis. In the present study, we performed single-cell RNA sequencing (scRNA-Seq) using the 10x Genomics platform to analyze the changes in murine adrenal transcriptome following systemic *C.albicans* infection. A total of 16 021 cells were categorized into 18 transcriptionally distinct clusters, representing adrenocortical cells, endothelial cells, various immune cells, mesenchymal cells, smooth muscle cells, adrenal capsule, chromaffin cells, neurons and glials. As the main cell component in the adrenal gland responsible for steroidogenesis, the adrenocortical cells dramatically diminished and were further grouped into 10 subclusters, which differently distributed in the infected and uninfected samples. Pseudo-time analysis revealed transitions of the adrenocortical cells from the initial normal states to active or dysfunctional states following systemic *C.albicans* infection *via* two trajectory paths. Endothelial cells in the highly vascularized organ of adrenal gland further proliferated following infection, with the upregulation of genes positively regulating angiogenesis and downregulation of protective genes of endothelial cells. Immune cells were also excessively infiltrated in adrenal glands of *C.albicans*-infected mice. Macrophages dominated the immune microenvironments in murine adrenal glands both before and after *C.albicans* infection, mediating the crosstalk among the steroid-producing cells, endothelial cells and immune cells within the adrenal gland. NLR family, pyrin domain containing 3 (NLRP3, encoded by *Nlrp3*) and complement receptor 3 (CR3, encoded by *Itgam*) were found to be significantly upregulated on the adrenal macrophages upon systemic *C.albicans* infection and might play critical roles in mediating the myeloid response. Meanwhile, the number and strength of the interactions between the infiltrating immune cells and adrenal resident cells were unveiled by cell-cell communication analysis to be dramatically increased after systemic *C.albicans* infection, indicating that the immune-adrenal crosstalk might contribute to the compromised functions of adrenal cells. Overall, our comprehensive picture of the murine adrenal gland microenvironment in systemic *C.albicans* infection provides deeper insights into the immune-adrenal cell communications during fungal sepsis.

## Introduction

Systemic fungal infection remains a major health threat leading to high morbidity and mortality rates in immunocompromised patients, including those who are receiving immune-suppressive drug treatment or chemotherapy, or with pre-existing infections or malignant diseases (1, 2). *Candida albicans* (*C. albicans*) is the most common opportunistic fungal pathogen that can cause life-threatening disseminated candidiasis and fugal sepsis (3). There are an estimated 400,000 *Candida* bloodstream infections each year worldwide, with a mortality rate of up to 50% (4).

The adrenal gland is a key component of the body stress system that can be affected by microbial infections. It is a part of the hypothalamic pituitary-adrenal (HPA) axis and the sympathetic adrenal medullary (SAM) system (5). Accordingly, the adrenal gland integrates two distinct anatomical regions, the steroid hormone-producing cortex which is regulated by the adrenocorticotropic hormone (ACTH), and the catecholamine-producing medulla which is under the control of the sympathetic nervous system (SNS) (6). The cortex comprises three separate zones, zona glomerulosa (zG), zona fasciculata (zF), and zona reticularis (zR). The syntheses of mineralocorticoids, glucocorticoids and adrenal androgens occur in the three cortical zones, respectively, each using specific enzymes. The chromaffin cells residing in the medulla secrete catecholamines (7). During stress, these hormones impact tremendously the functioning of all tissues, glands, and organs to help the body to restore homeostasis (8). However, around 20% of critically ill patients will develop some form of the adrenal gland insufficiency (AI), which may increase up to 60% in patients with septic shock, further raising the risk of mortality due to sepsis (5, 9, 10).

Sepsis, including fungal sepsis, is one of the extreme examples of severe, sustained physical stress caused by dysregulated systemic host inflammatory responses to microbial infections, leading to life-threatening multiorgan dysfunction (4, 5). A fast adrenal response is critical to survive this adverse condition (11). The adrenal gland microenvironment, where multiple direct and paracrine interactions among different cell types take place, influences the progression of sepsis and is influenced in turn (5, 12).

Over the past few years, some studies have been focusing on various aspects of the adrenal dysregulation induced by bacterial sepsis (5, 8, 12). A positive correlation was observed between the severity of sepsis and the degree of adrenal damage characterized by hemorrhages, apoptosis, immune cell infiltration, and lower response to ACTH test (13). And the sepsis-induced alteration in the adrenal microenvironment could directly influence the adrenal cells’ functions (12, 14). The adrenal recruitment of populations of immune cells and the immune-adrenal crosstalk in LPS-induced systemic inflammatory response syndrome (SIRS) or sepsis were also reported (5, 8, 12).

However, the adrenal gland stress response during fungal sepsis has been rarely reported. Therefore, we aimed to depict the landscape of the adrenal gland microenvironment in response to systemic fungal infections. As *C. albicans* is one of the most common pathogens of nosocomial bloodstream infections, in the present study, we constructed a murine model of systemic *C. albicans* infection and performed single-cell RNA sequencing (scRNA-Seq) using the 10x Genomics platform to better understand the complex adrenal microenvironment and the changes induced by *C. albicans* infection. Bioinformatics analyses identified seventeen cell types belonging to adrenocortical cells, adrenomedullary cells, endothelial cells, immune cells, and stromal cells. Significant shifts in the cell proportions and functions, as well as increased immune-adrenal communications after *C. albicans* infection were deduced from the single-cell transcriptomic information. Our study will provide a comprehensive picture of the murine adrenal gland microenvironment and bring deeper insights into the immune-adrenal crosstalk during fungal sepsis.

## Methods

### Murine model of systemic *Candida albicans* infection

Thirty 8-week-old female C57BL/6 mice weighing 20 ± 1g were randomly divided into two equal groups. All animals were housed under specific pathogen-free conditions. The reference strain *C. albicans* SC5314 was used for the systemic infection in a murine model. An inoculum of yeast cells was prepared at a concentration of 4 × 10^6^/mL in Normal saline (0.9% NaCl) and injected *via* the lateral tail vein into 8-week-old female Balb/c mice (0.1 mL per mouse, n=15). Control mice received only saline. At 48h after *C. albicans* infection, mice were sacrificed, blood were removed and both adrenal glands were collected for single-cell isolation or hematoxylin and eosin (H&E) staining. The kidneys and the adrenal glands were aseptically removed, weighed and homogenized in water, plated on yeast extract peptone dextrose (YPD) plates after serial dilutions, incubated at 30°C for 2 days, and colony forming units (CFU) were counted. Fungal burden was represented as CFU per gram of tissue. The animal work were approved by the Ethics Committee for Animal Use of the Peking University Health Science Center (Beijing, China), under the protocol number LA2020108.

### Histopathological analysis

Formalin-fixed and paraffin-embedded (FFPE) adrenal gland blocks were prepared, cut into 5 µm thick sections, baked onto slides at 60°C for 1 h, and then deparaffinized. Slides from the infected and control animal were stained with hematoxylin and eosin (H&E) or periodic acid-schiffs (PAS) following standard procedures and scanned by a NanoZoomer microscopic slide scanner (Hamamatsu Photonics, Hamamatsu, Japan).

### Single-cell isolation and transcriptome sequencing

Fresh adrenal gland tissues from each group were pooled and washed twice in ice-cold RPMI 1640 medium (GIBCO) containing 0.04% BSA (GIBCO), briefly chopped and then digested using collagenase I (0.2%; TermoFisher Scientific) at 37°C for 30 min, mixed upside down every 5-10 min, and then filtered through a 40-μm nylon mesh (BD). After centrifugation at 300 g, 4°C for 5 min, the cell pellet was resuspended in RPMI 1640/10% fetal bovine serum (FBS; GIBCO). After lysing the red blood cells with Red Blood Cell Lysis Solution (MACS) and removing dead cells with Dead Cell Removal Kit (MACS), the cells were counted and assessed for viability using Trypan blue staining on a Luna cell counter. Subsequently, single cells were captured and barcoded cDNA libraries were generated using the Chromium Next GEM Single Cell 3ʹGEM, Library & Gel Bead Kit v3.1 (10X Genomics) (15). After passing quality tests, all single-cell cDNA libraries (paired-end) were sequenced to 50,000 reads per cell on an Illumina Nova 6000 PE150.

### Data processing and cell clustering

Raw reads generated in high-throughput sequencing are processed with the CellRanger Single Cell software suite (https://support.10xgenomics.com) to conduct the preliminary quality control and obtain the absolute number of each transcript in a single cell. The low-quality cells were further filtered by using Seurat software package (16) based on the criteria of high-quality cells that the gene numbers and UMI of each cell are within the range of mean value ± twofold of the median absolute deviations (MAD), and the mitochondrial transcripts accounting for less than 20% of total counts. Double cells were removed by using DoubletFinder software (17). The batch effects were overcome by performing the mutual nearest neighbors (MNN) with the R package bachelor (18). The FindVariableGenes function in Seurat package was used to screen highly variable genes (HVGs). The expression profile of hypervariable genes was used for principal component analysis (PCA) for cell clustering, and the results were visualized in 2-dimensions by UMAP (nonlinear dimensionality reduction). The marker genes that are differentially up-regulated in each cell cluster relative to other cell groups were identified by using the FindAllMarkers function in Seurat package (16) and visualized by VlnPlot and FeaturePlot functions. The cell clusters were then annotated using SingleR package (18) based on ImmGen reference data set (19). To further refine the automated annotation, significantly upregulated transcripts and well-established cell type-specific markers curated from the literature (20–22) were cross-referenced (**Table S1**).

### Differentially expressed genes and pathway enrichment analysis

The FindMarkers function in Seurat package (16) was used to screen the differentially expressed genes (DEGs). Genes exhibiting fold changes (FC) ≥ 1.5 with P-values < 0.05 between cases and controls were defined as DEGs. The DEGs with log FC < 0 were considered as down-regulated genes, while the DEGs with log FC > 0 were considered as up-regulated genes. Gene Ontology (GO) (molecular function) and Kyoto Encyclopedia of Genes and Genomes (KEGG) pathway enrichment analyses of the DEGs were carried out using R based on the hypergeometric distribution test. Gene Set Variation Analysis (GSVA) (23) was performed using standard settings, as implemented in the GSVA package.

### Pseudo‐time analysis

Cells within the adrenocortical cluster were selected for pseudo-time analysis using the Monocle2 package (24). The importCDS function was employed to convert the raw counts from Seurat object into CellDataSet object. The differentialGeneTest function was used to derive DEGs from each cluster and genes with q-value < 1e-5 were selected to order the cells in pseudo-time analysis. Cell ordering was performed on these genes with orderCells function in an unsupervised manner with default parameters. The cell trajectories were then constructed after dimensionality reduction with the reduceDimension function. Differentially expressed genes were tracked over pseudo‐time by using the plot_genes_in_pseudo-time function.

### Cell-cell communication analysis

The CellPhoneDB (v2.0) was employed for cell-cell interaction analysis as described previously (25). Enriched receptor-ligand interactions were derived between two cell types where the ligand was expressed by one cell type and the corresponding ligand was expressed by the other cell type. Igraph and Circlize softwares were employed to show the networks of cell‐to‐cell communication.

## Results

### Systemic *C. albicans* infection induced remarkable pathological changes in murine adrenal glands

**Figure 1:**
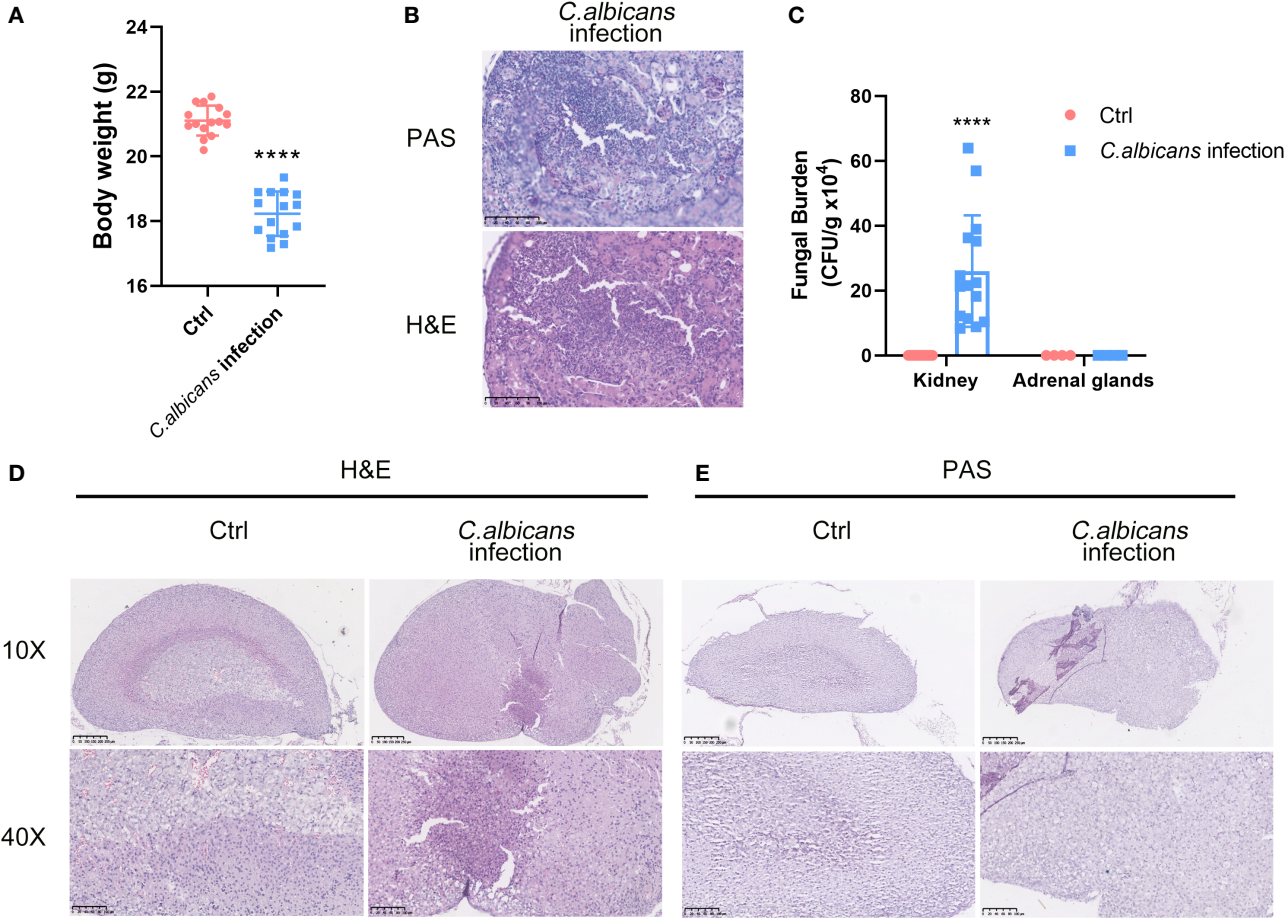
Murine model of systemic *C. albicans* and the histopathological changes in the adrenal glands. Female C57BL/6 mice (n=15) were inoculated with 4 × 10^5^ **(C)** *albicans* cells/mouse *via* the lateral tail vein. **(A)** The body weights of the infected animals and uninfected controls (Ctrl) before sacrifice at 48 h post infection. ****P<0.0001. **(B)** Representative PAS and H&E staining of kidneys from infected mice at 48 h post-infection. The pink filamentous stainings by PAS indicated the hyphae in the tissue. Magnification 400×. **(C)** The average fungal burdens in the kidneys and the adrenal glands of the infected animals and uninfected controls (Ctrl) were counted. ****P<0.0001. **(D)** H&E staining of the adrenal gland sections from the infected mouse and control. The boundary between the adrenal cortex and medulla became entirely blurred. The examples of hemorrhages, necrotic/apoptotic cells, and infiltrated immune cells were indicated by red, yellow, and black arrows, respectively. **(E)** PAS staining of the adrenal gland showed no obvious hyphae. Magnification 100× and 400×.

*C. albicans* is the major pathogen of nosocomial bloodstream infections in critically ill patients that leads to fungal sepsis with substantial morbidity and mortality. To depict the landscape of the adrenal gland microenvironment in response to systemic fungal infections, we performed single-cell RNA sequencing (scRNA-seq) using a murine model of systemic *C. albicans* infection. Female C57BL/6 mice were inoculated with 4 × 10^5^ C*. albicans* cells/mouse *via* the lateral tail vein and euthanized at 48 h post infection when the infected animals lost about 15% of their body weights as compared with uninfected controls (**Figure 1A**). As the primary target organ of *C. albicans*, the kidneys from infected mice were shown to contain a mass of hyphae by PAS staining (**Figure 1B**), with the average fungal burden being about 2.6 × 10^5^ CFU per gram of tissue (**Figure 1C**), which confirmed that all of the infected animals developed disseminated candidiasis. Adrenal glands were obtained at necropsy and one from each group was randomly selected as a representative for H&E and PAS staining. As shown in **Figure 1D**, remarkable pathological changes were found in the sections from the infected mouse, where the boundary between the adrenal cortex and medulla became entirely blurred, and diffuse mild hemorrhage was seen throughout. At high magnification, necrotic/apoptotic cells were observed in the cortex and immune cells were found to be heavily infiltrated. However, the adrenal glands were not the host sites of *C. albicans* as showed by PAS staining (**Figure 1E**) and CFU counts (**Figure 1C**).

### Single‐cell transcriptomic atlas unveiled diverse cell types in murine adrenal glands

**Figure 2:**
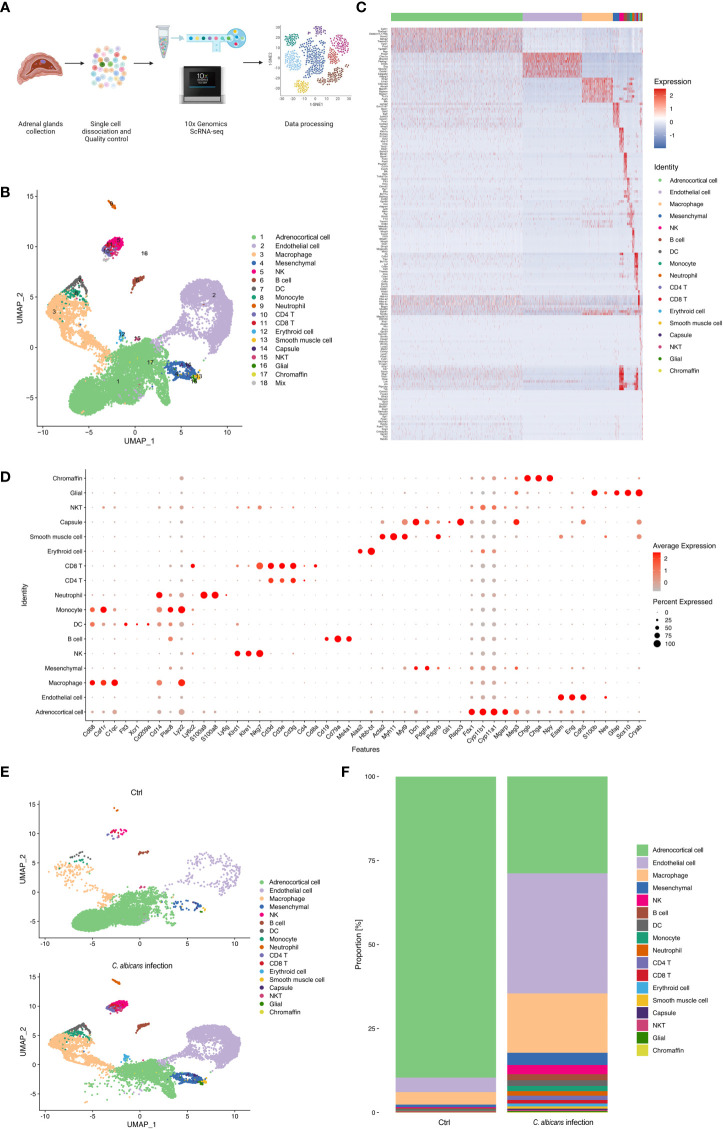
Single-cell RNA-seq analysis of the murine adrenal glands following systemic *C. albicans*. **(A)** Schematic of the single-cell RNA sequencing (scRNA-seq) workflow. **(B)** UMAP was used to clustering the cells in murine adrenal glands. Samples were pooled from 15 mice per group for analysis. Cell types were determined by using SingleR package based on immgenreference data set and well-established cell type-specific markers. **(C)** Heatmap showing expression of enriched markers genes for each cell type in scRNA-seq. **(D)** Bubble plot of the specific marker genes used for annotation of each cell type. **(E)** UMAP plots showing the cell clusters in the adrenal glands from the infected mice (bottom) and uninfected controls (top). And the proportion of each cell type in each group was shown in **(F)**.

We isolated single cells from the adrenal homogenates of mice at 48 h post infection and uninfected controls, and processed samples for scRNA-seq using the 10× Genomics Chromium platform. The whole workflow is shown in **Figure 2A**. After initial quality control with Cell Ranger and removing low-quality cells with Seurat, single-cell transcriptomes of a total of 16 021 cells (infected group 9 847 and uninfected control 6 174) were acquired (**Table S2**). All the scRNA-Seq data are deposited in the interactive cell browser https://ngdc.cncb.ac.cn/gsa. Based on hypervariably expressed genes, 18 transcriptionally distinct clusters of cells were identified by performing PCA dimensionality reduction and UMAP visualization (**Figures 2B**). The clusters were sequentially numbered according to the cell counts from more to less. Each cluster was then compared to the others to find unique gene signatures (**Table S3**) and the top 10 significantly up-regulated genes of each cluster were shown in the heatmap (**Figure 2C**). Well-established markers for known adrenal cell types were taken into account to annotate the cell clusters (**Figure 2D**).

The genes related to steroid hormone biosynthesis, *Fdx1*, *Cyp11b1*, *Cyp11a1*, *Meg3* and *Mgarp* were highly expressed in the cluster 1 (C1), implying that this cluster contained adrenocortical cells (**Figures S1A**). The endothelial markers *Esam*, *Eng*, and *Cdh5* were significantly enriched in the C2, defining them as endothelial cells (**Figures S1B**). High expression levels of *Cd68*, *Csf1r*, and *C1qc* defined C3 cells as macrophages (**Figure S1C**). The mesenchymal markers, *Dcn*, *Pdgfra*, and *Pdgfrb* were found abundant in C4, supporting they were mesenchymal cells (**Figure S1D**). C5 was identified as NK cells for the high expression of *Klrd1*, *Klre1*, and *Nkg7* (**Figure S1E**). C6 was marked by *Ms4a1*, *Cd79b*, and *Cd19* as B cells (**Figure S1F**). C7 was identified by the markers of dendritic cells including *Flt3*, *Xcr1*, and *Cd209* (**Figure S1G**). C8 was determined to be monocytes due to the high expression of *Plac8*, *Lyz2*, *Ly6c2* and *Cd14* (**Figure S1H**). C9 expressed high levels of *S100a8*, *S100a9* and *Ly6g*, and was thus recognized as neutrophils (**Figure S1I**). C10 and C11 were CD4+ and CD8+ T cells, respectively, because they highly expressed *Cd3d*, *Cd3e*, and *Cd3g*, as well as the corresponding markers, *Cd4* and *Cd8a* (**Figure S1J**). C12 expressed high levels of erythroid markers, *Alas2* and *Hbb-bt* (**Figure S1K**). C13 was considered as smooth muscle cells for the high levels of well-known markers, including *Acta2*, *Myl9*, and *Myh11* (**Figure S1L**). C14 was identified by *Gli1* and *Rspo3*, markers expressed in the adrenal capsule (**Figure S1M**). C15 showed high levels of both CD3 markers, *Cd3d*, *Cd3e*, and *Cd3g*, and NK markers *Nkg7*, and was identified as NKT cells. Neuron marker *Cryab* and glial markers *S100b*, *Nes*, *Gfap*, and *Sox10* were found enriched in C16 (**Figure S1N**). Chromaffin markers including *Chgb*, *Chga*, and *Npy* defined C17 as chromaffin cells (**Figure S1O**). And C18 was judged as a small group of mixed cells because they showed unspecific expression of multiple cell markers.

These results revealed that the murine adrenal gland microenvironment was highly heterogeneous and composed of diverse cell types. Following systemic *C. albicans* infection, the proportions of most cell types were largely changed as compared to the control group (**Figures 2E, F**), which might lead to the structural and functional damage of the adrenal gland.

### Adrenocortical cells dramatically diminished and underwent dysfunction during candidiasis

**Figure 3:**
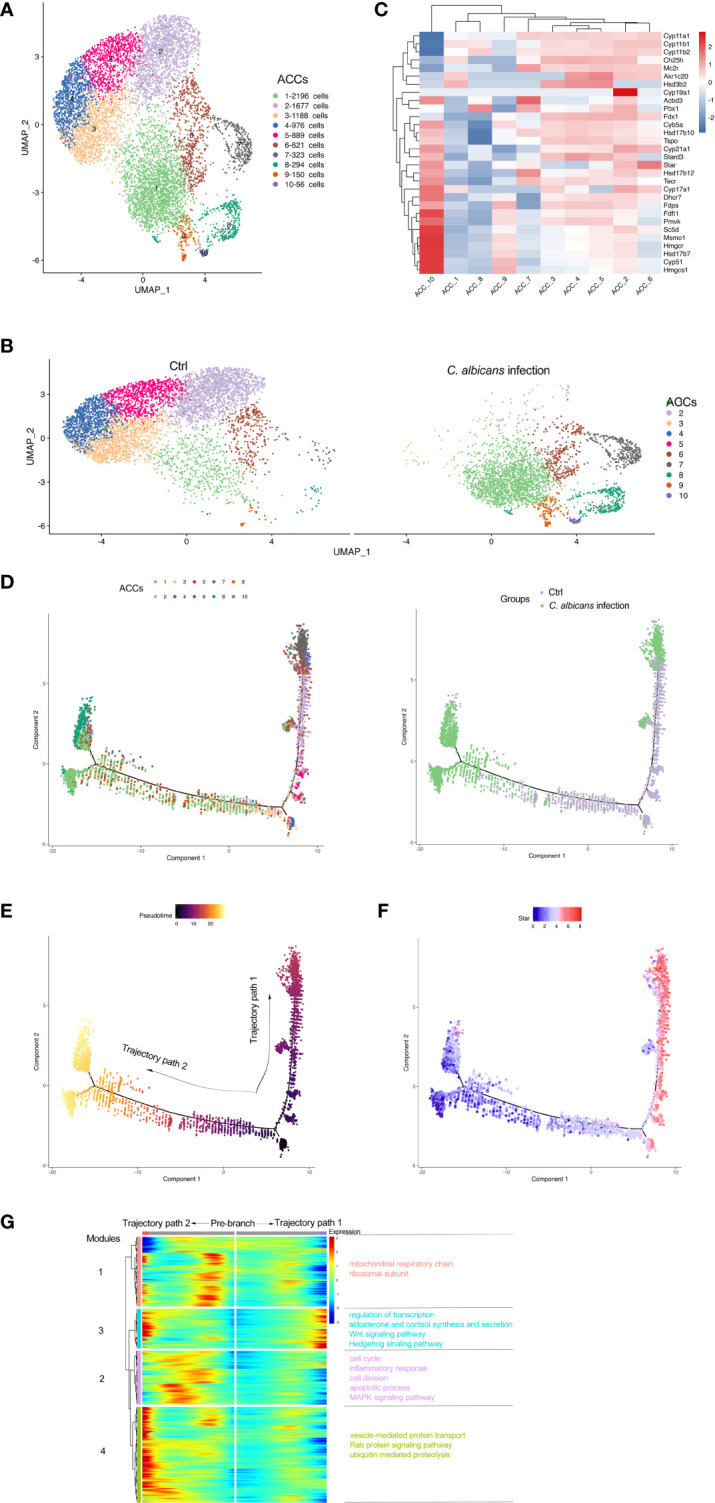
Subclusters and pseudo-time trajectory of adrenocortical cells in *C. albicans-*infected mice and uninfected controls. **(A)** Adrenocortical cells from both groups were pooled and subjected to subclustering on the UMAP plot. **(B)** The different distributions of the adrenocortical subclusters (ACCs) originating either from infected or uninfected animals shown by UMAP plots. **(C)** Heatmap of the expression patterns of the steroidogenesis associated genes across the 10 AACs. **(D)** The transitions of adrenocortical cells during candidiasis progression along the pseudo-time trajectory, shown by ACCs (left) and groups (right). **(E)** The pseudo-time trajectory in case the transition states corresponding to ACC3 and ACC4 were designated as the beginning. **(F)** Expression pattern of *Star* along the pseudo-time trajectory. Each point indicates the log-normalized expression level in a single cell. **(G)** Heatmap of different modules of DEGs along the pseudo-time trajectory. Representative significantly enriched GO and KEGG functional processes were shown.

As the main cell component in the adrenal gland responsible for steroidogenesis, adrenocortical cells constituted a vast majority (nearly 90%) of the total populations identified by scRNA-seq under the normal condition, while this proportion declined sharply to 28.8% after Systemic *C. albicans* infection (**Figure 2E, F**). Additionally, the distribution of this cell cluster on the UMAP plot changed dramatically, which prompted us to further divide them into 10 subclusters (Adrenocortical cluster ACC1 to ACC10) based on single‐cell gene expression profiles using UMAP (**Figure 3A**). As compared to the uninfected control, where AACs 2, 3, 4, and 5 made up the majority of the adrenocortical cells, infected adrenal cortex were dominated by AACs 1, 7, 8, 9, and 10 (**Figure 3B**).

The expression patterns of the steroidogenesis associated genes (7, 8) across the 10 AACs was then analyzed to functionally annotate the adrenocortical subsets (**Figure 3C**), and pseudo‐time reconstruction was carried out to explore the transitions of adrenocortical cells during candidiasis progression (**Figure 3D** and **S2A**). The percentage of the subclusters that were more enriched in the infected sample increased along the trajectory, validating the reliability of the trajectory analysis and determined the beginning of the trajectory path (**Figure 3E**). Expression levels of *Star* that encodes steroidogenic acute regulatory protein (StAR) that is involved in the cholesterol intracellular transport to initiate steroidogenesis (26) were found to coincide well with the transition states of adrenocortical cells (**Figure 3F**). The adrenocortical transition was initiated from ACC3 and ACC4 (low levels of *Star*) which represented the normal states with low to moderate levels of the steroidogenesis (**Figures 3C, D**). Through the intermediate states of ACC2, ACC5, and ACC6 (low to high levels of *Star*) that were characterized by the elevated levels of steroidogenesis, some cells finally reached the active steroid-producing states of ACC6 (high levels of *Star*, *Cyp11a1*, *Cyp11b1*, *Cyp11b2*, and *Cyp17a1* for corticosterone and aldosterone synthesis), ACC7 (high levels of *Star*, *Cyp11a1*, and *Cyp11b2* for aldosterone synthesis) and ACC10 (high levels of *Star*, *Cyp17a1*, and *Cyb5a* for androgen synthesis) (trajectory path 1) (7); while the other cells went through the intermediate active state of ACC6 and got to the incompetent states of ACC1, ACC8 and ACC9 (low levels of *Star*), characterized by very low levels of steroid biosynthesis (trajectory path 2) (**Figures 3C, D** and **S2A**).

We next investigated the dynamic transcriptional changes associated with transitional states and observed four different gene expression modules (**Figure 3G** and **Table S4**). GO and KEGG analyses (**Figures S2B–E**) of these dynamically expressed genes indicated that the expression of genes enriched in mitochondrial respiratory chain, cell cycle, and apoptotic process fluctuated, and finally dropped along trajectory path 1 while increased at the termination of trajectory path 2 (modules 1 and 2), suggesting that the cell fate of path 2 might be undergoing proliferation with attenuated steroidogenesis (27, 28) or apoptosis due to the alterations of mitochondrial oxidative phosphorylation or cell cycle progress (29). Wnt and Hedgehog signaling pathways were also enriched in module 3, which might be crucial for proper development and regeneration of the adrenal glands (30, 31). Genes of module 3 were involved in regulation of transcription and aldosterone and cortisol synthesis and secretion, which increased during candidiasis in both paths. Genes of module 4 increased first and then decreased in both paths and were enriched into vesicle-mediated protein transport and proteolysis. These results revealed that the regulation of protein expression at the transcription and post-transcription levels is critical for the adrenal response to *C. albicans* infection.

Overall, the adrenocortical cells dramatically diminished in number with functions partially impaired in the context of systemic *C. albicans* infection, which might be due to the increased cell death triggered by the inflammatory microenvironment in the infected adrenal glands.

### Endothelial cells massively proliferated following systemic *C. albicans* infection

**Figure 4:**
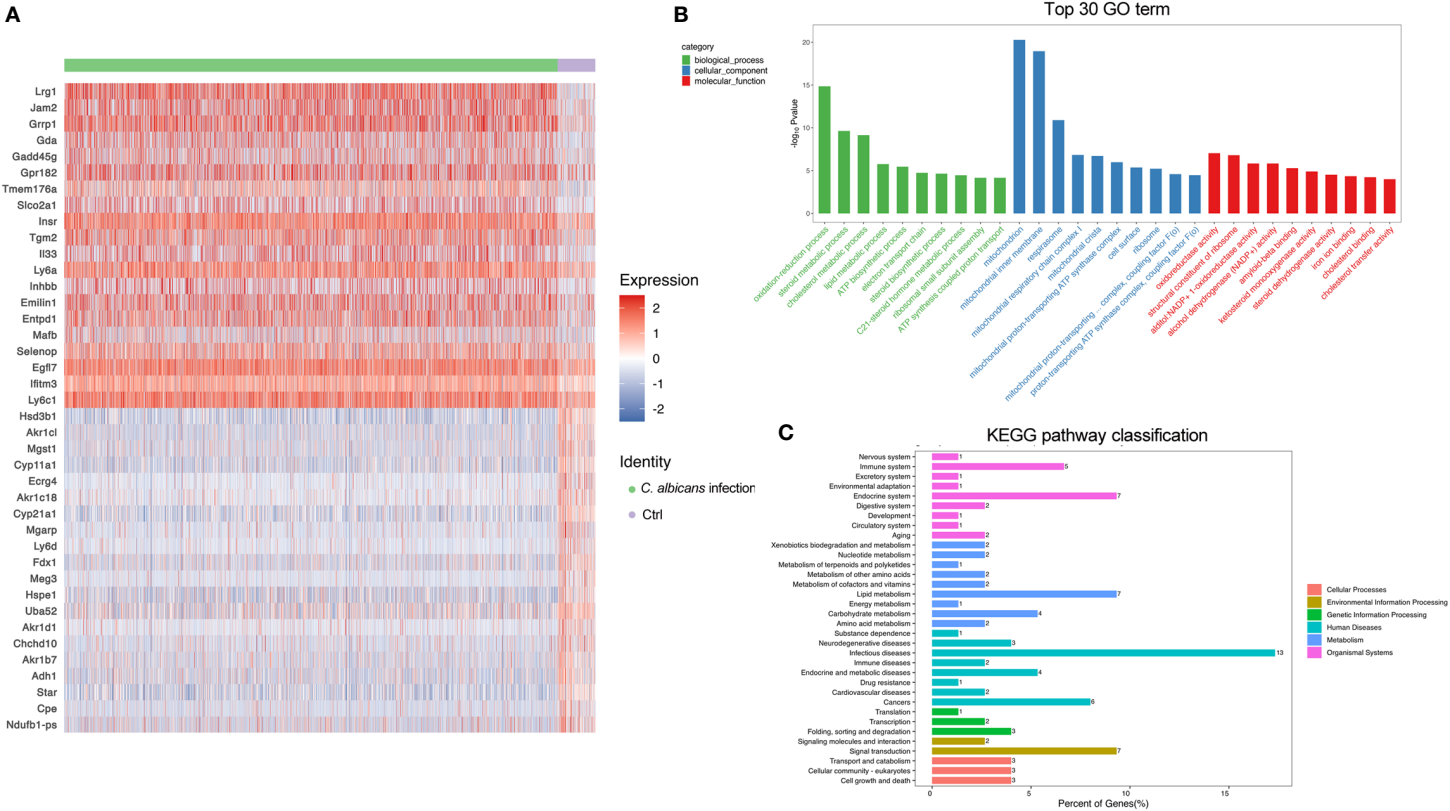
DEGs in endothelial cells following systemic *C. albicans*. **(A)** Heatmap of the top 20 DEGs between the infected and uninfected groups. **(B)** GO and **(C)** KEGG enrichment analyses of the DEGs.

The adrenal gland is a highly vascularized organ with a large number of endothelial cells lying closely to the steroid-producing cells in the adrenal cortex (32). As illustrated in **Figures 2E, F**, the endothelial cells were even more abundant after systemic *C. albicans* infection, consistent with the proliferative vasculature observed in the murine adrenal gland slices from the infected animals (**Figure 1D**). The DEGs were then identified (**Table S5**) to decipher the functional changes of the endothelial cells and a heatmap of the top 20 DEGs was generated (**Figure 4A**). GO and KEGG enrichment analyses of the DEGs revealed that upon *C. albicans* infection, endothelial cells were enriched for genes associated with oxidation-reduction process, lipid metabolic process, signal transduction and cell growth and death, which were involved most in infectious diseases (**Figures 4B, C**). Among the top 20 upregulated genes, there were the genes positively regulating endothelial cell proliferation and angiogenesis, such as *Lrg1*(32) and *Egfl7* (33, 34); the genes positively regulating MAPK cascade, such as *Gadd45g*(35) and *Insr*(36); the genes positively regulating cell migration and/or adhesion, such as *Insr*(37) and *Tgm2*(38); and *Il33* that positively regulates the secretion of immunoglobulin and chemokines and promotes macrophage activation (39). On the other hand, many of the top 20 downregulated genes were involved in the lipid metabolic process, including several aldo-keto reductase family members, such as *Akr1b7*, *Akr1c18*, *Akr1cl*, and *Akr1d1*, which may protect endothelial cells against damage elicited by lipid peroxidation (40). *Cpe* that encodes Carboxypeptidase E, a negative regulator of the Wnt signaling pathway (39), was also significantly downregulated. These results suggested that the adrenal vessels grew dramatically following systemic *C. albicans* infection, which might result from the enhanced adrenal blood flow induced by a number of neural and hormonal mechanisms (32) and raised chances for paracrine interactions among the different cell types within the adrenal gland. However, the endothelial function might be impaired under the oxidative stress caused by the infection.

### Immune cells were excessively infiltrated in adrenal gland following systemic *C. albicans* infection

**Figure 5:**
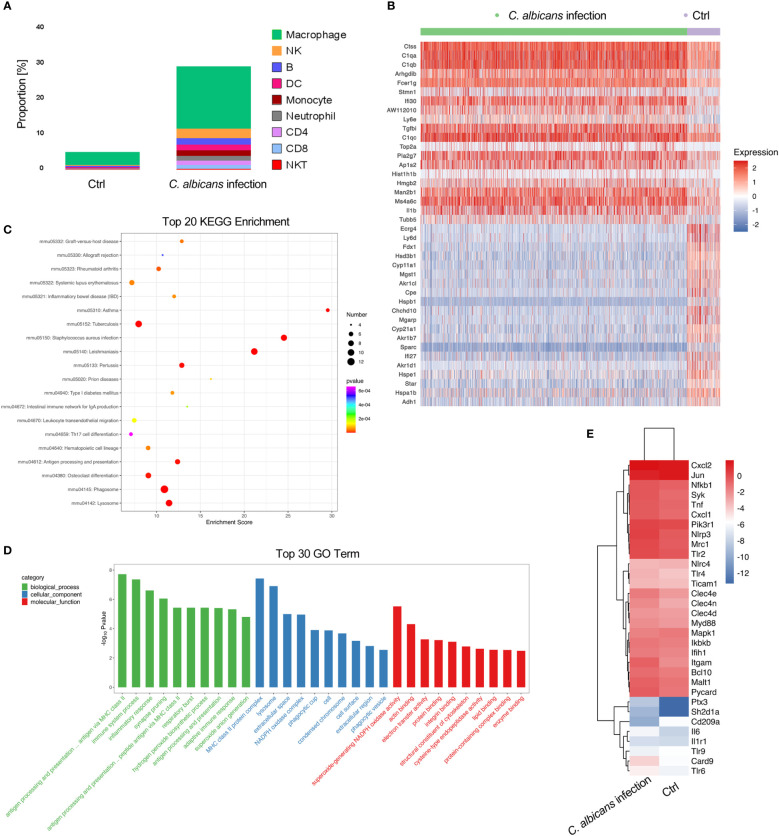
Infiltration of immune cells in the adrenal glands following systemic *C. albicans* infection and functional analysis of adrenal macrophages. **(A)** The proportions of immune cells within adrenal glands. **(B)** Heatmap of the top 20 DEGs in adrenal macrophages between the infected and control groups. **(C)** KEGG and **(D)** GO enrichment analyses of the DEGs in adrenal macrophages. **(E)** Heatmap showing the expressions of PRR genes involved in anti-fungal immunity and the related key signaling molecules in adrenal macrophages of infected mice and uninfected controls.

It has been documented that in the adrenal gland microenvironment under normal conditions, there are various immune cells such as macrophages, monocytes, dendritic cells, mast cells, and lymphocytes located in direct contact with adrenocortical, chromaffin and endothelial cells (41–44). They mainly participate in host defense mechanisms. In agreement with the reports, we observed a variety of immune cells including macrophages, NK cells, B cells, DCs, monocytes, neutrophils, CD4^+^ and CD8^+^ T cells, and NKT cells in murine adrenal gland by scRNA-seq (**Figure 2B**). In uninfected samples, the resident immune cells accounted for about 5% of all sequenced cells, of which over 70% were macrophages. Systemic *C. albicans* infection resulted in remarkable increases of all populations of immune cells within adrenal gland. The proportion of immune cells surged to almost 30% of the total adrenal cells, with macrophages remaining the majority. The percentage of neutrophils showed the largest increase of 4.8-fold, followed by CD8^+^ T, CD4^+^ T and NK cells, whose proportions increased 2.20, 1.96, and 1.95-fold, respectively (**Figures 2E, F** and **5A**).

We then analyzed the DEGs in macrophages to understand their functions in adrenal gland inflammation during candidiasis (**Table S6**). A heatmap of the top 20 DEGs was shown in **Figure 5B**. GO and KEGG analyses indicated that the antigen processing and presentation *via* MHC class II, phagocytosis, and NAPDH oxidase mediated superoxide generation were the most enriched processes and functions involving the upregulated genes (**Figures 5C, D**). Consistently, MHCII-related genes *H2-Ab1*, *H2-DMa*, *H2-DMb1*, and *H2-Eb1*, as well as genes that play essential roles in MHC class II-peptide complex formation such as *Ctss* and *Ifi30*(45, 46) were found among the significantly upregulated genes in macrophages recruited to the adrenal gland upon *C. albicans* infection. NADPH oxidase complex components such as *Cyba*, *Cybb*, *Ncf1*, *Ncf4*, and *Ncf2*, complement components *C1qa/b/c*, and *Fcer1g* that positively regulates phagocytosis were also upregulated (**Figure 5B**). These observations suggested that these adrenal macrophages may be involved in antigen presentation, phagocytosis and ROS production in the adrenal gland microenvironment in response to systemic *C. albicans* infection. *Pla2g7* and *ll1b* that encode pro-inflammatory mediators PAF-AH and IL-1β, respectively were also upregulated. Notably, IL-1β can stimulate adrenal hormone production (47, 48), and PAF-AH is a booster of vascular inflammation (49), implying that the adrenal macrophages may mediate the crosstalk among the steroid-producing cells, endothelial cells and immune cells within the adrenal gland.

On the other hand, the expressions of genes encoding the pattern recognition receptors (PRRs) and the related key signaling molecules that are involved in innate antifungal recognition in macrophage were specifically analyzed (50). The comparison between the infected sample and uninfected control demonstrated that most of the PRR genes such as *Clec4n*, *Clec4e*, *Tlr2*, *Tlr6*, *Nlrp3* and *Mrc1*, as well as the downstream signaling molecules including *Syk*, *Card9*, *Bcl10*, *Malt1*, and *Myd88* that play critical roles in anti-fungal immunity were upregulated in adrenal macrophages upon *C. albicans* infection (**Figure 5E** and **Figure S3**). NLR family, pyrin domain containing 3 (NLRP3, encoded by *Nlrp3*) was found to be the most significantly increased PRR on the macrophages, which recognizes *C. albicans* hyphae in mice (50). And ASC encoded by *Pycard* that forms the inflammasome with NLRP3 (51) was consistently upregulated. In addition to PRRs, complement receptor 3 (CR3, encoded by *Itgam*) which recognizes non-opsonized *C. albicans*(50) was also remarkably elevated. Therefore, it could be speculated that NLRP3 and CR3 are critical for mediating the myeloid response in the adrenal gland during candidiasis.

### Immune-adrenal interactions increased after systemic *C. albicans* infection

**Figure 6:**
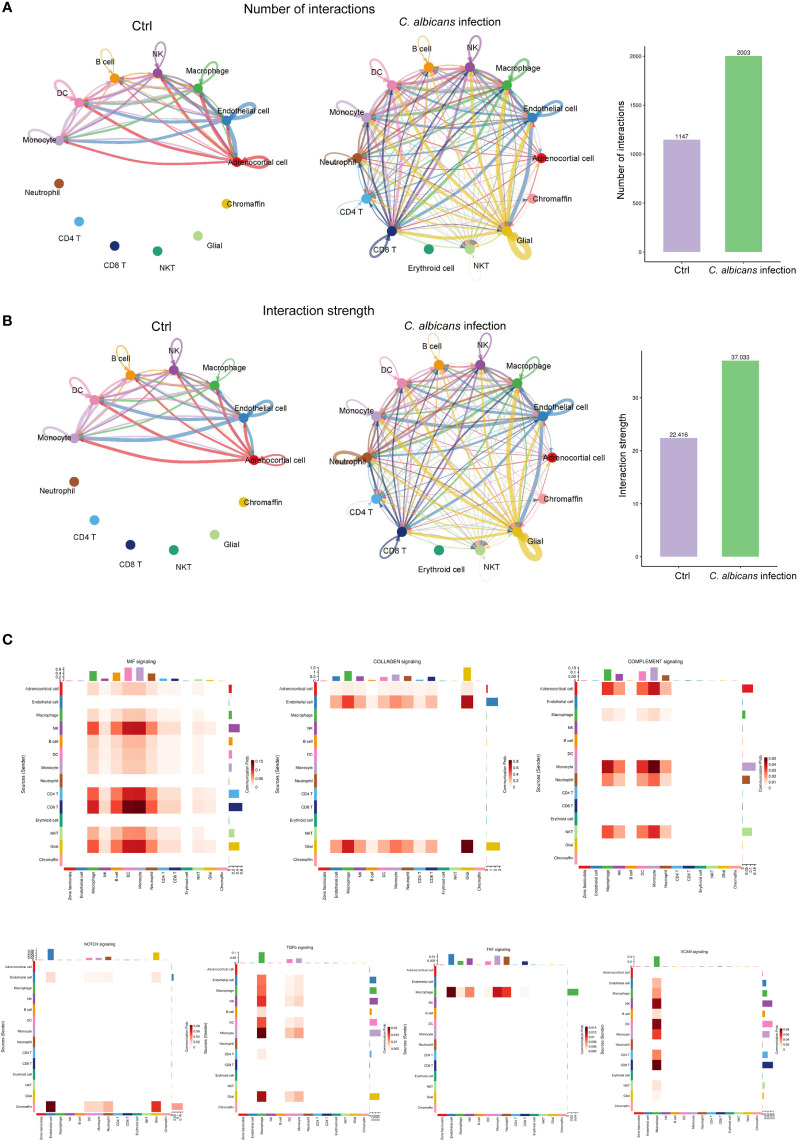
Cell-cell communication network analysis of multiple cellular connections in murine adrenal glands after systemic C. albicans infection. **(A)** The numbers and **(B)** the strength of the cellular interactions network in *C. albicans* infection and control groups. The nodes represent different cell types. The connecting line indicates the interaction between cell types, which color is the same as that of the ligand cell, and the arrow points to the receptor cells. The thickness of the line indicates the degree the number or strength of interactions. **(C)** Heatmaps showing the communication relationships at representative signaling pathways, with the X-axis and Y-axis representing the receptor cell and the ligand cell types, respectively. The top and right colored bars represent the sum of received and sent signals in each cell type, respectively.

Since the immune-endocrine interaction has been extensively studied in the circumstance of bacterial sepsis, we characterized the intercellular receptor-ligand pairs and molecular interactions among the various immune cells and adrenal resident cells following disseminated fungal infection by the CellPhoneDB algorithm (25). We found that both the numbers and the strength of the interactions between the infiltrating immune cells and adrenal resident cells were dramatically increased upon *C. albicans* infection (**Figures 6A, B**). Diverse receptor-ligand interplays were identified and the communication relationships at each signaling pathway level were visualized in form of heatmap by Cellchat (**Figure 6C**). Specifically, MIF signaling pathway (*Mif*, *Cd74*, *Cxcr4*, *Cd44*, *Cxcr2*) was found to most widely participate in the interactions among adrenocortical cells, glial/neuron cells and various immune cells. Collagen signaling pathway (*Col1a1*, *Col1a2*, *Col4a1*, *Col4a2*, *Col6a1*, *Cd44*, *Sdc4*, *Itga1*, *Itga9*, *Itgb1*, *Col9a2*, *Itga2*, *Itgav*, and *Itgb8*) contributed extensively to the interactions where adrenal cells including adrenocortical cells, endothelial cells, and glial/neuron cells produced ligands and immune cells expressed the corresponding receptors. Complement pathway (*C3*, *C4a*, *C3ar1*, *Itgam*, *Itgax*, and *Itgb2*) played roles in interactions between adrenocortical cells and immune cells. NOTCH pathway (*Dll4*, *Notch1*, *Notch2*, *Notch4*, and *Dlk1*) was significantly involved in the interactions between chromaffin cells and other cells. The communications between macrophages and adrenal cells as well as other immune cells involved TGFβ (*Tgfb1*, *Tgfbr1*, *Tgfb2*, and *Tgfbr2*), TNF (*Tnf*, *Tnfrsf1a*, and *Tnfrsf1b*), and VCAM (*Itga4*, *Itga9*, *Itgb1*, *Itgb7*, and *Vcam1*) pathways. These results indicate that the crosstalk between the immune and adrenal cells *via* diverse receptor-ligand signaling pathways may exert complex impacts on the adrenal insufficiency during candidiasis.

## Discussion

Single-cell RNA sequencing of the murine adrenal gland has been carried out very limitedly to map the normal mouse adrenal gland (20) or the developing adrenal gland to elucidate the cell origin and risk stratification of neuroblastoma (21, 22). As the pivotal component of the body’s stress system, the adrenal gland’s responses to severe pathological conditions e.g. sepsis should be more noteworthy. Over the last decades, fungal sepsis has emerged as a growing threat to human health with *C. albicans* being the most common pathogen in most clinical settings (52). Therefore, we were prompted to construct a single-cell resolution transcriptomic atlas of the murine adrenal glands following systemic *C. albicans* infection in the present study.

By cross-referencing the marker genes identified by the FindAllMarkers function in Seurat package and the established cell type-specific markers presented by the above studies (20–22), we annotated the 18 transcriptionally distinct clusters as adrenocortical cells, endothelial cells, various immune cells, mesenchymal cells, smooth muscle cells, adrenal capsule, chromaffin cells, neurons and glials, respectively, essentially consistent with the previous reports. Additionally, by identifying highly expressed genes that are differentially up-regulated in each cell cluster relative to other clusters, we might provide some new potential marker genes for cell populations in murine adrenal gland. For example, tetraspantransmembrane protein Cmtm5 (chemokine-like factor-like MARVEL-transmembrane domain containing protein 5) was found to rank first among the predominantly expressed genes in glial cells that used to be defined by S100b, Nes, GFAP, and Sox10 (**Figure 2C** and **S1N**) (21, 22). It has been reported that CMTM5 is highly enriched in oligodendrocytes and central nervous system (CNS) myelin, and is involved in the function of oligodendrocytes to maintain the integrity of CNS axons (53). Our data suggested for the first time that *Cmtm5* might also be used as a novel marker for glial cells in murine adrenal gland.

Rapid activation of the adrenal gland steroid production is a fundamental component of the stress response and is of utmost importance to survive sepsis. Both adrenal gland hyperactivation and insufficiency can be life-threatening (5, 12). The adrenal cortex is responsible for the biosynthesis of steroid hormones, including mineralocorticoids, glucocorticoids, and adrenal androgens, each in separate adrenal cortical zones using specific enzymes. They are Cyp11b2 essential for aldosterone production in zG, Cyp11b1 and Cyp17a1 essential for cortisol production in zF, and Cyp17a1 and Cyb5a essential for androgen production in zR (7). But above all, the steroidogenic acute regulatory protein (StAR) that is induced by ACTH stimulation (54) mediates the rate-limiting step in steroid biosynthesis, by transferring cholesterol, the substrate for all steroid hormones, from the outer to the inner mitochondrial membrane (26). Expression levels of Star were found to be most consistent with the trajectory of adrenocortical cells, which demonstrated two fates of these cells following systemic *C. albicans* infection. Some cells were activated for steroidogenesis, and the others were incompetent. Interestingly, from the expression heatmap of the steroidogenesis associated genes across the 10 subclusters of adrenocortical cells (ACCs), we could see that the three types of steroid-producing cells were mixed at the initial and intermediate states and could not be separated by subclustering based on gene expression pattern, as the ACCs 2-6 exhibited composite expressions of the signature enzyme genes. This might be explained by and in turn support the idea that steroidogenic cells of the different adrenocortical zones are thought to have a clonally-related origin (55) or be maintained by stem cell populations that share a common developmental origin (56). However, at the termination, cells producing different hormones were substantially assigned to different subclusters, such as ACC7 (high levels of *Cyp11b2* specific for aldosterone synthesis) and ACC10 (high levels of *Cyp17a1* and *Cyb5a* specific for androgen synthesis). Therefore, we could conclude that the massive changes in the transcriptome of adrenocortical cells were taken place during stress conditions, which made functionally different cells be distinguished apart.

By investigating the dynamic transcriptional changes associated with transitional states, we found that the signaling pathways including mitochondrial respiratory chain (module 1), cell cycle, and apoptotic process (module 2)were differentially regulated between the two trajectory paths; and that genes involved in aldosterone and cortisol synthesis and secretion increased during candidiasis in both paths (module3). The synchronous increase in the expression of both the steroidogenic enzymes and the mitochondrial oxidative phosphorylation system subunits induced by ACTH in adrenocortical cells might meet the metabolic needs of steroid hormone production (57). On the other hand, mitochondrial respiratory chain could modulate apoptosis in a context-dependent manner (29). There are also reports that adrenocortical cell proliferation during regeneration or following stimulations is accompanied by impaired steroidogenesis (27, 28, 58). It could be speculated that the mechanism underlying the adrenal malfunction might involve the asynchronous regulations of the steroidogenic and energy producing signaling pathways, and the cell apoptosis due to the dysregulated mitochondrial oxidative phosphorylation and cell cycle progress, and the lack of steroidogenic enzymes in a population of proliferative cells in the adrenal cortex.

The high vascularization in the adrenal gland supports the direct and paracrine interactions between different cell types within the adrenal gland microenvironment, but also increases its vulnerability for endothelial dysfunction and hemorrhage (32). We found that the endothelial cells remarkably increased in *C. albicans* infected-adrenal glands, with the genes positively regulating endothelial cell proliferation and angiogenesis upregulated, such as leucine-rich alpha-2-glycoprotein 1 (*Lrg1*). It was uncovered from diseased retinal microvessels and found to be mitogenic to endothelial cells and promote angiogenesis by modulating endothelial TGF-β signaling (59). Another upregulated gene, epidermal growth factor-like domain 7 (*Egfl7*) was also reported to mediate endothelial sprouting and the promotion of angiogenesis (33, 34). At the same time, some endothelium protective factors were found downregulated, including several aldo-keto reductase family members, such as *Akr1b7*, *Akr1c18*, *Akr1cl*, and *Akr1d1*, which may protect endothelial cells against damage elicited by lipid peroxidation (40). These findings suggested that the adrenal vascular hyperplasia occurred following systemic *C. albicans* infection, while the endothelial function might be impaired. As dysfunction of endothelial system is a hallmark of sepsis often leading to multiple organ damage, the genes mentioned above might be used as promising therapeutic target for controlling pathogenic angiogenesis and endothelial dysfunction during sepsis.

By scRNA-seq, we observed various immune cells in normal murine adrenal glands, including macrophages, NK cells, B cells, DCs, monocytes, neutrophils, CD4^+^ and CD8^+^ T cells, and NKT cells, with macrophages being the majority. Interestingly, a small cluster of erythroid cells were identified only after systemic *C. albicans* infection, which are immature erythrocytes with immunomodulatory functions produced by extramedullary erythropoiesis during anemia, pregnancy, or infections (60, 61). A recent study revealed that CD71^+^ erythroid cells were expanded in sepsis and can serve as independent predictors of the development of nosocomial infections and 30-day mortality (62). Their functions in adrenal glands during fugal sepsis will be of interest. However, mast cells that was reported to reside in adrenal glands (12) were undetectable in our study, probably due to their scarcity or intolerance to the single-cell isolation procedure. Although scRNA-seq provides the opportunity to identify rare cells and get insight into their functions, there is still room for improvement.

During bacterial sepsis, the adrenal glands are heavily infiltrated by circulating immune cells (5, 12). Consistently, our findings demonstrated that systemic *C. albicans* infection also triggered excessive infiltration of various populations of immune cells into adrenal gland. The percentage of neutrophils increased the most, while macrophages still makes up the vast majority of the immune cells. The functions of neutrophils are often impaired during sepsis. This results in their increased migration into random organs where they mediate tissue destruction and endothelial damage by increased ROS production, NETs formation and release of the active content of their granules (63, 64). In our study, the neutrophils were too rare in the uninfected sample to perform the DEG analysis. The effects of neutrophil accumulation in the adrenal gland during fungal sepsis need to be further elucidated.

Our data showed that macrophages dominated the immune microenvironment in murine adrenal glands. NLRP3 (NLR family, pyrin domain containing 3) was found to be the most significantly upregulated PRR on the macrophage after infection, which recognizes *C. albicans* hyphae in mice (50) and forms NLRP3 inflammasome with ASC (apoptosis-associated speck-like protein) to controls caspase-1 mediated cleavage of pro-IL1β (65). Therefore, it could be speculated that NLRP3 is critical for mediating the myeloid response in the adrenal gland during candidiasis. CR3 (complement receptor 3) which recognizes non-opsonized *C. albicans*(50) was also remarkably elevated. It can also mediate adhesion of inflammatory cells to the vascular endothelium, which might contribute to the accumulation of the macrophages within the infected adrenal glands (66). However, its upregulation has not been reported in the adrenal glands undergoing bacterial sepsis, so it might play some unique role in fugal sepsis. The C-type lectin receptors (CLR)-Syk adaptor CARD9, which has been shown to be crucial for antifungal immunity, was upregulated as well. CARD9 has been reported to be required for microglial pro-IL-1β transcription, inflammasome activation, and CXCL1 production which recruited CXCR2-expressing neutrophils in the fungal-infected brain (67, 68). In line with that, *Cxcl1* was also increased in adrenal macrophages following *C. albicans* infection, which might contribute to neutrophil recruitment to the adrenal microenvironment.

To elucidate the functional changes of the adrenal macrophages following systemic *C. albicans* infection, DEGs were analyzed. The antigen processing and presentation *via* MHC class II and phagocytosis were the most enriched processes and functions involving the upregulated DEGs, suggesting that these adrenal macrophages may engulf the infectious agents, bundle them inside the cell with MHCII, and then present them to the nearest lymph nodes (69). The upregulation of genes enriched in NAPDH oxidase mediated superoxide generation suggested an increased production of ROS that might contribute to the adrenal damage (70).

Moreover, the complement components C1qa/b/c and pro-inflammatory mediators PAF-AH and IL-1β were also found overexpressed by adrenal macrophages upon *C. albicans* infection. C1q is the defining component of the classical pathway of complement cascade, which is crucially involved in the pathogenesis of sepsis. Especially, the complement anaphylatoxin C5a leads to adverse effects and induces apoptosis in adrenomedullary cells during sepsis (71, 72). C1q has also been described to bind to apoptotic cells and opsonize the cell debris for effective phagocytosis (73). PAF-AH is a booster of vascular inflammation (49). IL-1β has been well documented to be capable of stimulating adrenal hormone production and inducing apoptosis in adrenocortical cells (47, 48). These secretory products enable the adrenal macrophages to mediate the crosstalk among the hormone-producing cells, endothelial cells and immune cells within the adrenal gland. On the other side, IL33 was found to be upregulated in the endothelial cells. It may function as an alarmin that acts on the immune system after endothelial cell damage during infection. Once passively released during cell necrosis or tissues destruction, it can promote the activation of many immune cells including macrophage (74). The immunomodulatory effects of adrenal hormones have also been well documented. For instance, glucocorticoids could exert both inflammatory and anti-inflammatory actions (5, 75). These findings suggested that there were complex communications between immune cells and other cell types in the adrenal gland microenvironment during systemic *C. albicans* infection.

Although the immune-endocrine interaction has been extensively studied for decades (6), there is still no single-cell resolution interaction map. By dissecting the receptor-ligand interaction network, we identified several signaling pathways central to the immune-adrenal crosstalk. For instance, Macrophage migration inhibitory factor (MIF) and its receptors CD74, CXCR4, CD44 and CXCR2 were found to be widely involved in the interactions among various types of cells in the adrenal gland. MIF is an important constituent of the host response to stress and infection and is the first mediator. It is released from adrenocortical cells and immune cells upon stimulation with glucocorticoids, and acts to counter-regulate the inhibitory effect of glucocorticoids on inflammatory cytokine production (76). Complement pathway (*C3*, *C4a*, *C3ar1*, *Itgam*, *Itgax*, and *Itgb2*) also played roles in interactions between adrenocortical cells and immune cells. C3 was detected to be highly expressed by adrenocortical cells, which matched the upregulation of the iC3b receptor CR3 (CD11b/CD18) on macrophages after *C. albicans* infection. Their binding can facilitate phagocytosis, oxidative burst, and downstream inflammation (77). These immune-adrenal crosstalk would exacerbate the adrenal gland inflammation and contribute to adrenal exhaustion characterized by decreased adrenal steroidogenesis.

Collectively, we present here a comprehensive picture of the murine adrenal gland microenvironment during systemic *C. albicans* infection by single-cell RNA transcriptomics analysis. Dramatic changes in the numbers, proportions and functions of multiple cell types in adrenal gland were identified and the immune-adrenal communication networks were dissected. Thus, our study provides deeper insights into the adrenal gland stress response during fungal sepsis.

## Data availability statement

The data presented in the study are deposited in the National Genomic Data Center - Genome Sequence Archive repository, accession number CRA007150 (https://bigd.big.ac.cn/gsa/browse/CRA007150).

## Ethics statement

The animal study was reviewed and approved by ethics committee for Animal Use of the Peking University Health Science Center (Beijing, China).

## Author contributions

TL acquired funding, conceived this study, and drafted, edited and revised the manuscript. RL acquired funding, supervised this study, and revised the manuscript. KZ constructed the murine model, collected samples, performed histopathological analysis, and created some figures for and edited the manuscript. YH performed parts of the data analysis and created some figures for the manuscript. All authors contributed to the article and approved the submitted version.

## Funding

This work was supported by grants from the National Natural Science Foundation of China (82071850) and(82030095).

## Conflict of interest

The authors declare that the research was conducted in the absence of any commercial or financial relationships that could be construed as a potential conflict of interest.

## Publisher’s note

All claims expressed in this article are solely those of the authors and do not necessarily represent those of their affiliated organizations, or those of the publisher, the editors and the reviewers. Any product that may be evaluated in this article, or claim that may be made by its manufacturer, is not guaranteed or endorsed by the publisher.

## Supplementary material

Table S1 Previously reported cell type-specific markers for annotation of the cells in murine adrenal glands.

Table S2 Cell statistics of scRNA-seq before and after QC.

Table S3 The marker genes for each cell cluster identified with the FindAllMarkers function.

Table S4 The DEGs of different modules along the pseudo-time trajectory in adrenlcortical cells.

Table S5 The DEGs in the endothelial cells.

Table S6 The DEGs in macrophages.

Figure S1 Expression levels of specific markers for each cell type are plotted onto the UMAP. **(A)** Adrenocortical cells; **(B)** Endothelial cells; **(C)** Macrophages; **(D)** Mesenchymal cells; **(E)** NK cells; **(F)** B cells; **(G)** Dendritic cells; **(H)** Monocytes; **(I)** Neutrophils (Figure S1I). **(J)** CD4+ and CD8+ T cells; **(K)** Erythriod markers; **(L)** Smooth muscle cells; **(M)** Adrenal capsule; **(N)** Neurons and glial cells; **(O)** Chromaffin cells.

Figure S2
**(A)** Group display of pseudo-time trajectory of adrenocortical cell subclusters (AACs). Each AAC is displayed separately on the trajectory. **(B–E)** GO and KEGG analyses of modules 1-4 of DEGs along the pseudo-time trajectory.

Figure S3 The violin plots showing the expressions of the key genes encoding the pattern recognition receptors (PRRs) and the related key signaling molecules that are involved in innate antifungal recognition in macrophages from the infected mice (Ca) and uninfected controls (Ctrl).
