# Supplementary figures and images for: Single-cell atlas of murine adrenal glands reveals immune-adrenal crosstalk during systemic Candida albicans infection
Source: Front Immunol. 2022 Nov 1;13:966814. doi: 10.3389/fimmu.2022.966814 (PMC9664004; doi:10.3389/fimmu.2022.966814)

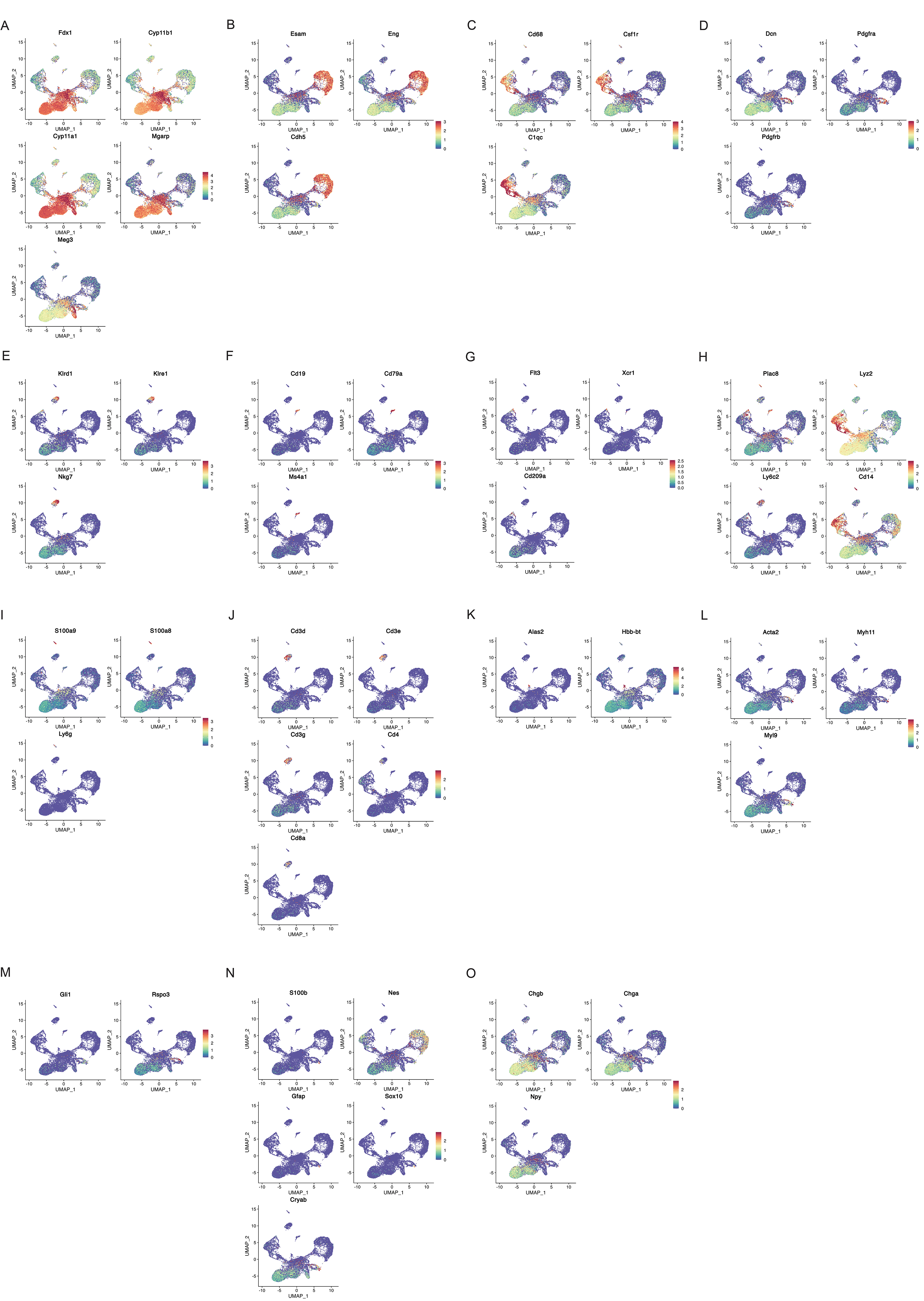

Supplement: Figure S1 — Expression levels of specific markers for each cell type are plotted onto the UMAP. (A) Adrenocortical cells; (B) Endothelial cells; (C) Macrophages; (D) Mesenchymal cells; (E) NK cells; (F) B cells; (G) Dendritic cells; (H) Monocytes; (I) Neutrophils (Figure S1I). (J) CD4+ and CD8+ T cells; (K) Erythriod markers; (L) Smooth muscle cells; (M) Adrenal capsule; (N) Neurons and glial cells; (O) Chromaffin cells. [file Image_1.jpeg]

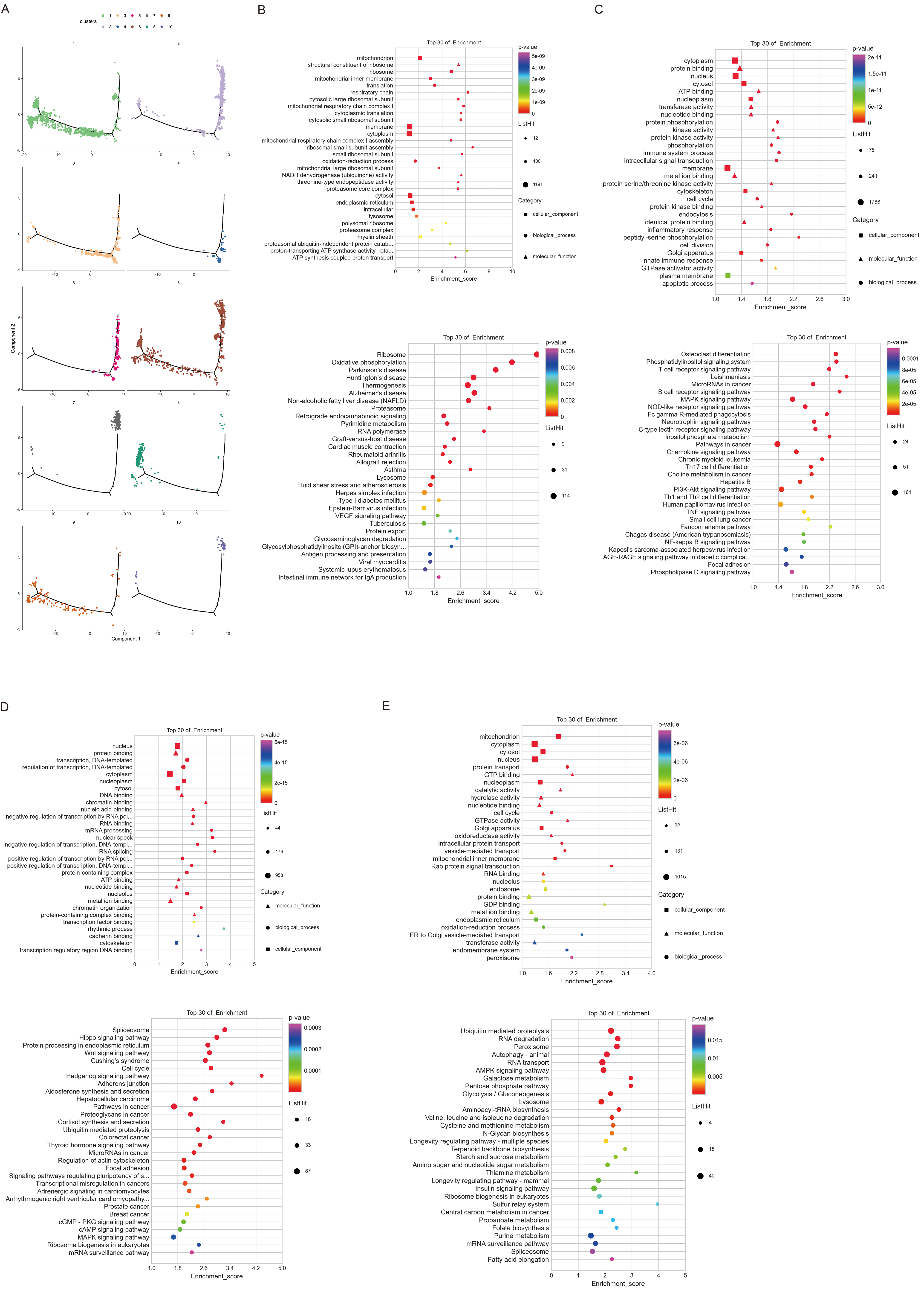

Supplement: Figure S2 — (A) Group display of pseudo-time trajectory of adrenocortical cell subclusters (AACs). Each AAC is displayed separately on the trajectory. (B–E) GO and KEGG analyses of modules 1-4 of DEGs along the pseudo-time trajectory. [file Image_2.jpeg]

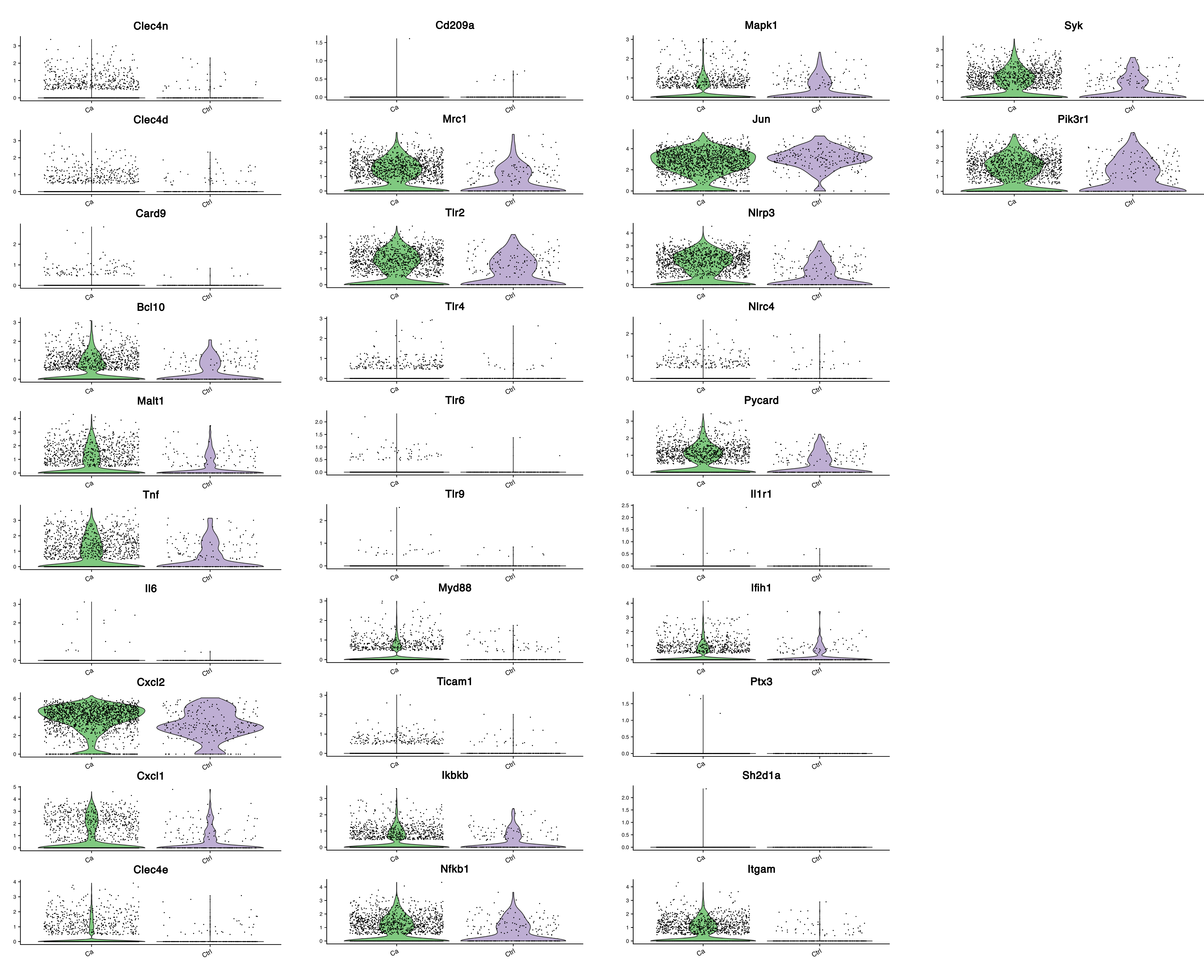

Supplement: Figure S3 — The violin plots showing the expressions of the key genes encoding the pattern recognition receptors (PRRs) and the related key signaling molecules that are involved in innate antifungal recognition in macrophages from the infected mice (Ca) and uninfected controls (Ctrl). [file Image_3.jpeg]
