# Supplementary material for: Single-cell atlas of murine adrenal glands reveals immune-adrenal crosstalk during systemic Candida albicans infection
Source: Front Immunol. 2022 Nov 1;13:966814. doi: 10.3389/fimmu.2022.966814 (PMC9664004; doi:10.3389/fimmu.2022.966814)
Supplement: Supplementary file 10 [file DataSheet_1.docx]

**TABLE S1** | Previously reported cell type-specific markers for annotation of the cells in murine adrenal glands.

**TABLE S2** | Cell statistics of scRNA-seq before and after QC.

**TABLE S3** | The marker genes for each cell cluster identified with the FindAllMarkers function.

**TABLE S4** | The DEGs of different modules along the pseudo-time trajectory in adrenlcortical cells.

**TABLE S5** | The DEGs in the endothelial cells.

**TABLE S6** | The DEGs in macrophages.

**FIGURE S1** | Expression levels of specific markers for each cell type are plotted onto the UMAP. (A) Adrenocortical cells; (B) Endothelial cells; (C) Macrophages; (D) Mesenchymal cells; (E) NK cells; (F) B cells; (G) Dendritic cells; (H) Monocytes; (I) Neutrophils (Figure S1I). (J) CD4+ and CD8+ T cells; (K) Erythriod markers; (L) Smooth muscle cells; (M) Adrenal capsule; (N) Neurons and glial cells; (O) Chromaffin cells.

**FIGURE S2** | (A) Group display of pseudo-time trajectory of adrenocortical cell subclusters (AACs). Each AAC is displayed separately on the trajectory. (B-E) GO and KEGG analyses of modules 1-4 of DEGs along the pseudo-time trajectory.

**FIGURE S3** | The violin plots showing the expressions of the key genes encoding the pattern recognition receptors (PRRs) and the related key signaling molecules that are involved in innate antifungal recognition in macrophages from the infected mice (Ca) and uninfected controls (Ctrl).
